# Editorial: Epidemiological characteristics of ocular trauma globally and their clinical implications

**DOI:** 10.3389/fmed.2026.1758737

**Published:** 2026-01-29

**Authors:** Mary Jae Abellana Felizarta, Mohammad Soleimani

**Affiliations:** 1 University of North Carolina at Chapel Hill, Chapel Hill, NC, United States; 2 Department of Ophthalmology, University of North Carolina at Chapel Hill, Chapel Hill, NC, United States

**Keywords:** ophthalmic trauma, ocular trauma, exogenous endophthalmitis, global epidemiology, intraocular foreign body (IOFB), open globe injury, parasitic ocular infections

Ocular trauma is a leading cause of monocular blindness worldwide, with an estimated 55 million eye injuries occurring each year and 750,000 cases requiring hospitalization. The effects are debilitating, with ocular injuries leaving approximately 1.6 million blind and almost 19 million with unilateral blindness or low vision (1). The burden of ocular trauma poses an economic problem as well, with annual direct and indirect costs for ocular injuries being conservatively estimated at $5 million with a loss of 60 work years (2). Ocular trauma exhibits variability across geographic locations, socioeconomic status, age, and cultural practices. However, there is no single international multicenter registry in ophthalmic trauma, though such a registry could enhance understanding by providing large real-world datasets that more accurately reflect the population. Currently, 37 countries are participating in The International Globe and Adnexal Trauma Epidemiology Study (IGATES) ophthalmic trauma registry, but engagement across countries is not universal (3). The studies included in this Research Topic: *Epidemiological Characteristics of Ocular Trauma Globally and Their Clinical Implications* support the need for international collaboration, by consolidating data on ocular trauma inpatients across several regions and populations, in order to provide clinicians with evidence-based insights for ocular trauma prevention, prognostication, and management.

Pelletier et al. provide a foundational contribution to understanding the global epidemiology of ocular trauma, highlighting its disproportionate impact on low- and middle-income countries (LMICs). This narrative review consolidates epidemiological characteristics across geographic regions, socioeconomic contexts, and vulnerable populations—including children, women, and older adults. Although the risk of ocular trauma varies across different age groups in LMICs, males aged 20–40 years old have a higher incidence of ocular trauma due to occupational hazards, as they are more likely to work in high-risk professions such as construction, mining, and agriculture. Furthermore, rapidly industrializing countries have shown an increase in manufacturing and construction activities, contributing to the increase in work-related ocular trauma. It is crucial to note that LMICs are affected by structural determinants that decrease access to ophthalmic care, such as limited specialty services, overburdened facilities, and cultural or geographic barriers, all of which can delay treatment and lead to poorer visual acuity outcomes. Open globe injuries were prevalent across studies, which require prompt surgical management. However, there is an international variance in open globe injury surgical skills, as many countries neither define standards for competence nor require a minimum number of surgeries for graduating ophthalmology residents (4). The creation of internationally developed tools such as Ophthalmology Surgical Competency Assessment Rubric (OSCAR) can standardize training in open globe management for ophthalmic residents, which can address gaps in access to specialty ophthalmic services in LMICs.

Building on this global framework, this original research study uses the Global Burden of Disease data to assess the burden of intraocular foreign bodies (IOFB) injuries on pediatric populations. Unintentional injuries, specifically foreign bodies, are a leading cause of ocular injury globally (5). It was determined that the burden of IOFBs predominantly affects individuals aged 15–19 years old, irrespective of sex, likely due to heightened curiosity and engagement outside of supervised settings that children aged 0–4 years old would typically experience. This study concluded that although there was a reduction in the disease burden of IOFB injury from 1990 to 2021, the BAPC predictive model hypothesizes that after 2021, there will be an increase in incidence and disability-adjusted life years (DALYs) in individuals aged 0–19 years old, raising a major public health concern and the need for globally standardized approaches toward IOFB treatment and management.

The heterogeneity of ocular trauma mechanisms makes timely diagnosis and treatment by an ophthalmologist essential, as early intervention can improve the prognosis and salvage visual acuity (6). A study conducted by Mansouri et al. reports that vitrectomy for ocular trauma involving the posterior segment improved visual acuity outcomes, with no significant differences in prognosis between early and delayed vitrectomies. Rather, more favorable visual outcomes were associated with the absence of afferent pupillary defect, ocular trauma scores, presenting visual acuity, and the zone of injury (7). However, this included original research study examined 29 patients with exogenous endophthalmitis caused by metallic IOFBs and concluded that early vitrectomy was crucial in reducing infection and inflammation. Although intravitreal broad-spectrum antibiotics were effective in treating metal-induced endophthalmitis, adjunct early vitrectomy within 2 h of symptom exacerbation was associated with fewer complications, thus leading to favorable outcomes for any secondary surgeries.

Under-documented mechanisms of ocular trauma further complicate prevention and management, as seen in this multicenter original research study of animal-induced ocular injuries in Iran. Insects were the most common culprits, typically causing periorbital soft-tissue trauma, while birds, domestic pets (dogs and cats), and large animals, such as equines, each resulted in distinct injury patterns: anterior segment damage, adnexal trauma, or combined anterior and posterior involvement, respectively. The authors emphasize early and appropriate first-contact management, advising primary care providers to avoid mechanical removal of foreign bodies, prioritize ocular protection, and arrange urgent ophthalmology referral, supplemented by antibiotic prophylaxis and immunosuppressive therapy.

Finally, the case report by Lu et al. highlights the rare but significant nature of ocular parasitic infections. Ophthalmomyiasis is a parasitic infection of the eye most caused by *Oestrus ovis* dipteran larvae, an obligate parasite that typically inhabits the nasal passages and sinuses of sheep and goats. However, this case report documents a 35-year-old male with no reported livestock exposure residing in Bulgaria who presented with external ophthalmomyiasis. Although Bulgaria's temperate climate and the patient's lack of animal contact would typically make *O. ovis* ocular infestation improbable, first-instar larvae were identified and mechanically removed with sterile forceps. Treatment involved systemic and topical antibiotics, anti-inflammatory therapy, and repeated ocular irrigation, which eventually restored his corneal integrity and vision. While conclusions are limited by the single-case nature of this report, the authors recommend early recognition, mechanical removal, and follow-up examination within 24–48 h to prevent ocular damage. Another case report adds an important nuance to the mechanical removal of parasitic ocular infections, describing a 59-year-old male with subconjunctival dirofilariasis of the *Dirofilaria immitis* worm who presented with diurnal photophobia and temporal conjunctival injection. During slit-lamp examination and extraction under the operating microscope, the *Dirofilaria immitis* worm exhibited significant light sensitivity, making extraction difficult due to its movement (8). This clinical point should be considered during ocular parasitic or larval mechanical removal.

Ultimately, this Research Topic examines epidemiological characteristics of ophthalmic trauma globally, with the purpose of underscoring the critical need for international collaboration in order to improve prognostic and diagnostic classification systems, preventive measures, medical education, and management approaches for ocular trauma worldwide.
